# Assessing the validity and reliability of the 10-item Persian version of the perceived stress scale in post-surgery patients

**DOI:** 10.3389/fpsyt.2024.1402122

**Published:** 2024-06-04

**Authors:** Hamid Sharif-Nia, Erika Sivarajan Froelicher, Esmail Hoseinzadeh, Omolhoda Kaveh, Reza Fatehi, Poorya Nowrozi

**Affiliations:** ^1^ Psychosomatic Research Center, Mazandaran University of Medical Sciences, Sari, Iran; ^2^ Department of Nursing, Amol Faculty of Nursing and Midwifery, Mazandaran University of Medical Sciences, Sari, Iran; ^3^ Department of Physiological Nursing, School of Nursing, and Department of Epidemiology and Biostatistics, School of Medicine, University of California San Francisco, San Francisco, CA, United States; ^4^ Department of Nursing, Faculty of Medical Sciences, Gorgan Branch, Islamic Azad University, Gorgan, Iran; ^5^ Department of Nursing, Sari Faculty of Nursing and Midwifery, Mazandaran University of Medical Sciences, Sari, Iran; ^6^ Student Research Committee, Mazandaran University of Medical Sciences, Sari, Iran

**Keywords:** perceived stress, validity, reliability, psychometrics, Iran

## Abstract

**Introduction:**

The 10-item Perceived Stress Scale (PSS-10) is commonly used to measure stress levels in postoperative patients, as research shows that high levels of stress can affect postoperative outcomes. By using the PSS-10, healthcare providers can understand patients’ psychological well-being before and after surgery, helping improve recovery and overall health. This study focuses on assessing the reliability and validity of the 10-item Persian version of the PSS (PSS-10-P) in postoperative patients.

**Methods:**

In a methodological study conducted between October to December 2023, a sample of 400 patients who had undergone surgery in 17 Shahrivar Hospital, Amol, Iran were selected using a convenience sampling method. The PSS-10 scale utilized in the study was translated, and its psychometric properties were evaluated through assessments of construct validity, including exploratory (n = 200) and confirmatory (n = 200) factor analysis, convergent validity, and discriminant validity. Furthermore, the study examined the internal consistency of the scale to ensure its reliability.

**Results:**

The mean age of the participants was 44.38 (SD= 13.49) years. The results of exploratory factor analysis with Promax rotation extracted two factors accounting for 83.82% of the variance comprising 10 items. After necessary modifications during CFA, the final model was approved. As for reliability, the Cronbach’s alpha, CR, and MaxR for all constructs were greater than 0.7, demonstrating good internal consistency and construct reliability.

**Conclusion:**

According to these results, the Persian version of PSS-10 has a valid structure and acceptable reliability. This scale can be used by health professionals in many ways.

## Introduction

Surgery is one of the situations that can cause stress and anxiety in patients (1). Surgery is a deliberate change in the anatomical structures of the body for the purpose of creating comfort, alleviating or eliminating pathological processes, and repairing traumatic injuries (2). Surgery is a stressful experience for patients and their families due to the physical and psychological stress it causes. The fear of the unknown, loss of control, and potential risk of harm all contribute to this anxiety-inducing situation (3, 4). Several studies demonstrated that physical stress, psychological stress, stress due to hospitalization, self-care stress, and spiritual stress are the main sources of stress experienced by patients who are candidates for various surgery that result in enhancing the risk of complications, morbidity, and mortality (5–11). A situation is stressful when it is perceived by a person as threatening and uncontrollable and that person does not have the necessary resources to face the situation (12). Previous studies indicate that the statistics for anxiety and stress in patients undergoing surgery are significant (13–16); therefore, assessment and appropriate interventions are needed (17–20). The skills that an individual develops throughout their life, particularly cognitive and emotional skills that are acquired when individuals are facing stressful situations, play a crucial role in their ability to manage stress (21).

There are a variety of tools available for assessing stress levels. One commonly used scale is the Perceived Stress Scale (PSS), which measures an individual’s perceived level of stress. The original version of the PSS was developed by Cohen et al. in 1983 and consisted of 14 items (22). PSS10- item and PSS 4- item are shorter forms of the PSS scale. Psychometric properties of the above scale have been done in different countries among the numerous participants including Italian precarious workers (23), Czech general adult population (24), Chinese cardiac patients who smoke (25), patients with Multiple Sclerosis in the USA (26), German adults (27), Arabic Version among women Emeriti students (28), Brazilian pregnant women (29), Chinese adults during the COVID-19 Pandemic (30), Ecuadorian university students (31), Korean patients with chronic diseases (32), and in different languages such as the Swedish (33) and France versions (34).

The 10-item version has been used in most of the studies, and on the other hand, the results of numerous studies also showed that the psychometric properties of PSS-10 are higher than they are in the PSS-14 and PSS-4 (23–26, 35); for this reason, we chose to assess the validity and reliability of the 10-item version in our study. Only one study in Iran assessed the factorial validity of the 14-item version of this scale in cancer patients (36), but psychometric properties have not been thoroughly investigated and the reliability was not reported. Also, two studies conducted in Iran were among different samples (chronic headache and asthmatic patients) (37, 38). Therefore, the purpose of this study was to examine the psychometric properties of Persian versions of the PSS-10 (PSS-10-P) in postoperative patients.

## Methods

This methodological cross-sectional study was carried out between October to December 2023. Patients from 17 Shahrivar Hospital, Amol, Iran, were recruited for this study.

### Inclusion and exclusion criteria

The inclusion criteria for participants in the study were: being at least 18 years old, being able to communicate in Farsi and being literate, volunteering to participate, and being hospitalized in hospital wards after surgery. Exclusion criteria included cognitive disorders, presence of mental illness, reduced level of consciousness, heart diseases such as uncontrolled unstable angina and uncontrolled severe arrhythmia, limited activity due to severe physical diseases or cerebrovascular diseases, pregnancy, cancer and malignancies, other neurological diseases or rheumatoid arthritis, drug addiction, and drug dependencies, and not having a mental illness such as schizophrenia or anxiety disorder.

In their 1999 study, MacCallum and et al. recommended a minimum sample size of 200 cases for psychometric studies (39). To ensure the construct validity, it was necessary to invite 400 individuals to participate (two different samples for construct validity). The participants were approached in the hospital wards following their surgeries. After receiving a thorough explanation of the study’s objectives, they were invited to voluntarily participate and complete the 10-item Perceived Stress Scale (PSS-10) questionnaire.

### The original version of the scale

The 10-item PSS developed by Cohen deals with perceived stress and includes two associated areas. The scale questions include positive and negative aspects of stress. Response options are scored on a 5-point Likert scale (0-4) with the following scores: 4: never, 3: rarely, 2: sometimes, 1: often, 0: always. It should be noted that items 4, 5, 7, and 8 were positively calculated. A total score of 13 represents a normal stress level, but scores of 20 or higher represent high stress levels that require therapeutic intervention (35).

### Translation

The scale was translated from English to Persian by the established translation protocols (40). Two skilled translators proficient in both English and Persian independently translated the PSS-10 into Persian. An expert panel, consisting of the authors of this article (EF, and RF), and two professional translators, carefully reviewed and combined the two translations to produce a Persian version of the PSS-10. Following this, a Persian-English translator was hired to translate the PSS-10-P back into English. The panel of experts then reviewed and approved this PSS-10-P final version. Then the panel of experts compiled and compared the results of the back-translation with the original scale to detect any differences and similarities between the original scale and the back-translated version. All items are translated into Persian and back-translated into English without any required modifications.

### Normal distribution, outliers, and missing data

Skewness ( ± 3) and kurtosis ( ± 7) were used to individually investigate the univariate distribution of data. Also, the multivariate normality distribution was assessed by the Mardia coefficient of multivariate kurtosis (<8). Mahalanobis d-squared (p < 0.001) was used to determine whether there were any multivariate outliers (41). The missing data were assessed using multiple imputations, and the average participant response was used to replace the missing data (42).

### Construct validity

To assess the construct validity, the original dataset consisting of 400 cases was randomly split into two datasets, each containing 200 cases. The first dataset was subjected to Maximum Likelihood Exploratory Factor Analysis (MLEFA) with Promax rotation and Kaiser normalization, along with exploratory graph analysis methods to identify the underlying factor structure. The Kaiser-Meyer-Olkin (KMO) measure exceeding 0.8 and the significance of Bartlett’s test of sphericity (p < 0.01) were considered to confirm the suitability and relevance of the data for conducting factor analysis. Parallel analysis was utilized to determine the number of factors (43). The Eigenvalues of more than 1, communalities of more than 0.2, and factor loadings of more than 0.5 were also used for the factor extraction (44). Eigenvalues (λ) are calculated as the sum of squared factor loadings (SSL) across all items (k) for each factor. This value indicates the proportion of variance in each item that can be accounted for by the analysis. To determine the percentage of total variance explained by a factor, the Eigenvalue is divided by the total number of items (43). The MLEFA was performed using SPSS version 27.

In the next step, the factor structures obtained from MLEFA were analyzed and confirmed by conducting Confirmatory Factor Analysis (CFA) based on the second random dataset (n = 200) using AMOS version 27. The following model fit indices were used to assess the model fit: Comparative Fit Index (CFI), Normed Fit Index (NFI), Goodness of Fit Index (GFI), Relative Fit Index (RFI), and Incremental Fit Index (IFI) was > 0.9; that of Root Mean Square Error of Approximation (RMSEA) was < 0.08; and for Minimum Discrepancy Function divided by degrees of freedom (CMIN/DF) < 3 was considered good (45).

### Convergent and discriminant validity

Also, convergent and discriminant validity were evaluated. For convergent validity, composite reliability (CR) should be greater than 0.7, and Average Variance Extracted (AVE) should be greater than 0.5 for each construct. Fornell and Larcker (46) stated that for psychological constructs, if AVE is less than 0.5, but CR is more than 0.7, the convergent validity can be considered acceptable.

For discriminant validity, this study used the heterotrait-monotrait ratio (HTMT) of the correlations criterion, where the HTMT ratio between all constructs should be less than 0.85 to achieve discriminant validity. Discriminant validity is a measure of how distinct a concept is from others in a framework. It is important for construct validity. The HTMT ratio is used to evaluate discriminant validity, calculated by comparing correlations between constructs. A value of 1 indicates perfect discriminant validity, while a value close to 1 suggests a problem. A high HTMT ratio indicates a lack of discriminant validity (47).

### Reliability

The Cronbach’s alpha, McDonald’s omega, average inter-item correlation coefficient (AIC), Composite Reliability (CR), and Maximal Reliability (MaxR) were calculated to estimate the internal consistency and construct reliability. If the α, Ω, CR, and MaxR were greater than 0.7 and AIC values of 0.2 to 0.4 were interpreted as acceptable internal consistency (48).

### Perceived stress score

Descriptive statistics were employed to calculate the mean score of the PSS-10. Additionally, an independent samples t-test was conducted to evaluate differences between the groups of men and women for the scale.

## Results

### Demographic characters

The mean age of the participants was 44.38 (SD= 13.49) years. Among the 400 participants, 152 (46.1%) were women and 178 (53.9%) were men. The demographic characteristics of the participants are listed in **Table 1**.

**Table 1:** Demographic characteristics of participants (n = 400).

Variables		Mean (SD)
**Age**		44.38 (13.49)
**Variables**		**N (%)**
**Gender**	Male	209 (52.25)
	Female	191 (47.75)
**Marital Status**	Single	81 (20.25)
	Married	319 (79.75)
**Education level**	High school	164 (41.00)
	Diploma	156 (39.00)
	Undergraduate	74 (18.50)
	Postgraduate	6 (1.50)
**Surgical history**	Yes	115 (28.75)
	No	285 (71.25)
**Type of surgery**	Tibia fracture	20 (6.1)
	Knee fracture	7 (2.1)
	Ankle fracture	12 (3.6)
	Femoral fracture	26 (7.9)
	Knee arthroplasty	5 (1.5)
	Forearm fracture	16 (4.8)
	Wrist fracture	13 (3.9)
	Elbow fracture	9 (2.7)
	Humorous fracture	11 (3.3)
	Laparotomy	58 (14.5)
	Appendectomy	60 (15.0)
	Cholecystectomy	43 (10.75)
	Hernia	25 (6.25)
	Chest tube placement	24 (7.3)
	Spinal surgery	72 (21.8)

### The results of MLEFA

The results of MLEFA with Promax with Kaiser Normalization rotation using the first random dataset (n = 200) extracted two factors accounting for 83.82% of the variance comprising 10 items. The none of items were removed from the original version. Moreover, the results of the KMO (0.919) and Bartlett’s test of sphericity (*p* < 0.001, Chi-square= 6734.747, *df* = 45) showed the sampling was adequate and appropriate for conducting the factor analysis. The detailed results of the MLEFA are shown in **Table 2**.

**Table 2:** The result of MLEFA on the two factors Persian version of the Perceived Stress Scale (n = 200).

Factor	Items	Factor loading	h^2^	λ	% Variance
Lack of stress management	**Q_1_.** In the last month, how often have you been upset because of something that happened unexpectedly?	0.972	0.966	4.961	49.61%
	**Q_2_.** In the last month, how often have you felt that you were unable to control the important things in your life?	0.964	0.969		
	**Q_3_.** In the last month, how often have you felt nervous and “stressed”?	0.953	0.939		
	**Q_10_.** In the last month, how often have you felt difficulties were piling up so high that you could not overcome them?	0.867	0.861		
	**Q_9_.** In the last month, how often have you been angered because of things that were outside of your control?	0.866	0.859		
	**Q_6_.** In the last month, how often have you found that you could not cope with all the things that you had to do?	0.823	0.834		
Coping with stress	**Q_4_.** In the last month, how often have you felt confident about your ability to handle your personal problems?	0.938	0.917	3.421	34.21%
	**Q_5_.** In the last month, how often have you felt that things were going your way?	0.934	0.934		
	**Q_7_.** In the last month, how often have you been able to control irritations in your life?	0.926	0.947		
	**Q_8_.** In the last month, how often have you felt that you were on top of things?	0.901	0.925		

h^2^, Communalities; λ,Eigenvalue.

### The results of CFA

**Figure 1:**
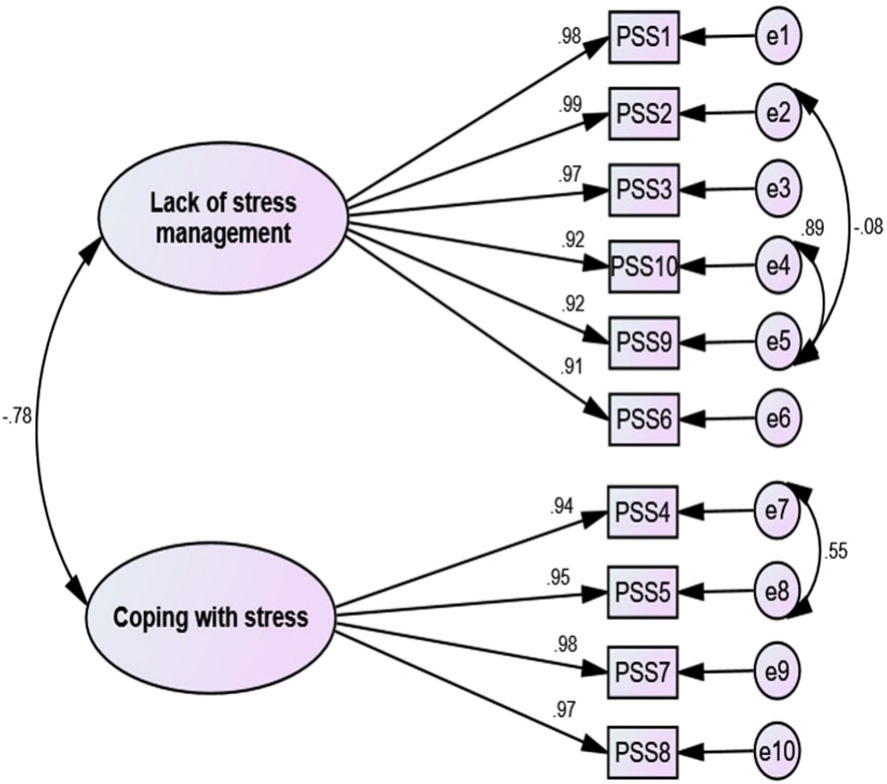
The results of the CFA and factor loading.

The CFA was conducted to confirm and validate the factor structure obtained from MLEFA using the second random dataset (n = 200). The results showed that the data fit the model well as evidenced by (*χ* ^2^(132) =218.505, *p* < 0.001, *χ* ^2^/*df* = 1.655, CFI = 0.978, IFI = 0.978, TLI = 0.969, SRMR = 0.067, RMSEA (90% C.I.) = 0.040 [0.043, 0.059]). The **Figure 1** shows the results of the CFA final model.

### Convergent and discriminant validity and reliability

The results showed that AVE for factors “lack of stress management and coping with stress” were greater than 0.5, indicating good convergent validity. As for discriminant validity, the results of the HTMT ratio were lower than 0.85, demonstrating good discriminant validity for all constructs. So results showed that the correlation between factor one and two (0.785). Cronbach’s alpha, McDonald’s omega, CR, and MaxR for all factors were greater than 0.7, and AIC was more than 0.2, demonstrating good internal consistency and construct reliability (**Table 3**).

**Table 3:** The results of the convergent validity and construct reliability (n = 200).

Factors	α	Ω	CR	MaxR	AVE	AIC
**Lack of stress management**	0.984	0.983	0.981	0.990	0.898	0.913
**Coping with stress**	0.982	0.982	0.979	0.984	0.921	0.931

α, Cronbach’s alpha; Ω, McDonald's omega.

### Perceived stress score

In the overall population, the mean score for PSS-10 was 20.14 (SD = 13.40, 95% CI: 18.69, 21.59) which 193 (48.25%) patients showed a high level of stress (perceived stress score greater than 20). Furthermore, there were no significant differences (*p* = 0.575) in PSS-10 scores between men (20.58, SD = 12.78) and women (19.69, SD = 14.12).

## Discussion

Perceived stress is commonly defined as the perception of stressful events encountered within a defined timeframe. Given the significant impact of stress on health (49), the primary objective of the current investigation was to assess the psychometric properties of the PSS-10 in Iranian surgical patients. The results of this research revealed that the PSS-10 exhibited acceptable factor structure, validity, and reliability.

The study found that the Persian version of the 10-item Perceived Stress Scale (PSS-10-P) had 10 items divided into two categories: lack of stress management and coping with stress. These two factors explained 83.82% of the variance in stress levels among Iranian surgical patients. In line with the objective of maximizing variance in factor analysis, reported variances of 56.23% (50) and in two other studies reported variances of 56.8% (49) and 61.9% (51).

The first factor of the PSS-10-P was designated as “Lack of stress management”. The term “stress management” is commonly utilized and appears to possess a well-defined definition (52). Inadequate stress management is when someone doesn’t have good strategies to deal with stress, which can harm their physical, mental, and emotional health. Symptoms can include headaches, fatigue, and more. If not managed, it can lead to distress and feeling overwhelmed. Effective stress management involves skills like problem-solving, time management, and relaxation techniques to navigate stress and maintain well-being (53). Interventions aimed at stress management encompass a range of techniques that offer opportunities for personal development (54). The focus on stress management is crucial to safeguard individuals’ physical and emotional well-being, as well as their capacity to function effectively in their daily lives. It is imperative to recognize stress and implement strategies to manage it effectively to mitigate the adverse repercussions it may engender (55). Lack of stress management means not having the right tools to deal with stress, which can have negative impacts on mental and physical health. It can lead to anxiety, depression, physical pain, and overall reduced quality of life. Without proper stress management, individuals may struggle with stressful situations and face long-term consequences on their health and functioning (56). Clinical studies have demonstrated that patients who exhibit a predefined adaptive stress response during surgery tend to experience superior recovery outcomes compared to those who do not (57). The items associated with the first factor underscore the importance for healthcare professionals to identify instances of inadequate stress management in patients. By imparting stress management techniques to patients, healthcare providers can help prevent the detrimental impacts of stress on surgical patients and can facilitate their swift recovery.

The second factor of (PSS-10-P) was denoted as “Coping with stress”. Coping, as defined, refers to the mental strategies and behavioral responses employed to manage stressful situations, whether they originate internally or externally. This term specifically pertains to purposeful, conscious decisions made with the aim of alleviating or enduring stress (58). Also Managing stress involves actively and intentionally addressing challenges that can trigger negative emotions. This includes utilizing strategies such as meditation, counseling, and engaging in hobbies to effectively manage and decrease stress levels. Coping with stress does not entail completely avoiding stress, but rather learning to handle it healthily to enhance overall well-being and resilience (56). The experience of preoperative stress induced by anesthesia and surgery prompts patients to employ coping mechanisms in an endeavor to regain a sense of emotional control. When coping is supported by specific techniques or external assistance, its efficacy may be enhanced (59). Consequently, nurses can play a pivotal role in alleviating the stress experienced by surgical patients by identifying their existing strengths in stress management and augmenting these capabilities through the application of stress coping strategies.

The CFA revealed that the data did not align well with the model, a finding consistent with previous studies (49, 60). Based on the analysis of construct validity and reliability, there is insufficient evidence to justify the utilization of two distinct sub-scales within the current population. The study’s outcomes also indicate that the items on this scale exhibit robust divergent and convergent validity. Divergent validity signifies complete segregation between constructs, while convergent validity is evident when elements of a construct are closely semantically related and account for variance (61).

Furthermore, the internal consistency coefficients of the scale dimensions demonstrate that the items within each factor exhibit substantial internal correlations, aiding in the clarification and measurement of a broader concept. Essentially, the components of each dimension effectively represent and assess a specific concept. Given the existing cross-cultural gap in health outcomes research (50), utilizing the PSS-10-P in the Persian culture with appropriate cultural variables can facilitate the identification of perceived stress among surgical patients and inform necessary interventions to mitigate it.

### Clinical and nursing implications

Validating the Persian Version PSS-10-P for postoperative patients is essential for nurses to accurately assess stress levels and customize care plans accordingly. This validation process enables nurses to deliver personalized interventions, address specific stressors, and enhance emotional resilience to facilitate smoother recoveries. By integrating the PSS-10-P into clinical practice, nurses can offer comprehensive care that acknowledges the influence of psychological factors on postoperative recuperation, ultimately fostering patient-centered care and enhancing outcomes for Persian-speaking populations.

### Limitations

Two limitations of the current study may introduce bias and subjectivity into the results. Therefore, there is a need for more robust clinical trials with longer-term follow-up to validate the findings and enhance their clinical significance.

### Future research

It can provide valid data for understanding the prevalence of this problem and investigating the impact of different interventions to reduce it. It is recommended that in future studies, other psychometric properties such as the responsiveness, sensitivity, and reliability (stability) of this scale, be investigated in diverse populations of both patients and healthy individuals who speak Farsi.

## Conclusion

The EFA results revealed the extraction of two factors, with ten items., which collectively account for 83.82% of the total variance in perceived stress among Iranian surgical patients. The findings affirm the appropriateness of employing the Persian iteration of the PSS-10-P as a dependable and valid instrument for assessing perceived stress in postoperative patients Healthcare professionals can effectively employ the PSS-10-P to educate patients on stress reduction strategies, thereby facilitating quicker post-surgical recovery and minimizing the likelihood of complications.

## Data availability statement

The raw data supporting the conclusions of this article will be made available by the authors, without undue reservation.

## Ethics statement

The Ethics Committee of Mazandaran University of Medical Sciences (Sari, Iran) gave its approval to this study (Ethics code: IR.MAZUMS.REC.1402.599). The participants were given a thorough explanation of the study’s goals and methods, as well as assurances that their participation was entirely voluntary. The researcher gave the scale to the patient to complete by themselves. The studies were conducted in accordance with the local legislation and institutional requirements. The participants provided their written informed consent to participate in this study.

## Author contributions

HS: Formal analysis, Supervision, Writing – original draft, Writing – review & editing. ES: Writing – review & editing. EH: Writing – original draft, Writing – review & editing. OK: Writing – original draft, Writing – review & editing. RF: Writing – original draft, Writing – review & editing. PN: Data curation, Writing – original draft.
